# Neoadjuvant FOLFIRINOX followed by Chemoradiotherapy for Middle and Lower Rectal Cancer

**DOI:** 10.31557/APJCP.2020.21.6.1717

**Published:** 2020-06

**Authors:** Amr Sakr, Mamdouh Elsherbeiny, Rabab Abdel Moneim, Saeed Shaaban, Moustafa Aldaly

**Affiliations:** 1 *Kasr Al-Ainy Center of Clinical Oncology and Nuclear Medicine (NEMROCK), Faculty of Medicine, Cairo University, Egypt. *; 2 *Clinical Oncology Department, Faculty of Medicine, Beni Suef University, Egypt. *

**Keywords:** Rectal cancer, FOLFIRINOX, neoadjuvant chemotherapy, sphincter preservation

## Abstract

**Objective:**

Neoadjuvant concomitant chemoradiotherapy followed by surgical resection is the standard of care in the treatment of rectal cancer. We are investigating the value of adding combination chemotherapy oxaliplatin, irinotecan, leucovorin and fluorouracil (FOLFIRINOX) before neoadjuvant chemoradiotherapy.

**Methods:**

Forty-one patients with middle and lower rectal cancer were included. FOLFORINOX were given every 2 weeks over 2 months (4 cycles) followed by concomitant chemoradiotherapy (CRT). Surgery was done 6-8 weeks after CRT and then adjuvant 4 months of FOLFOX or XELOX were given. The primary end point was sphincter preservation rate.

**Results:**

All patients received the four cycles of neoadjuvant chemotherapy FOLFORINOX, 38 patients completed CRT and only 29 patients underwent surgery. 32 patients were available for assessment (29 patients who underwent surgery and three patients who refuse surgery because of no evidence of disease by endoscopy, imaging and biopsy). Sphincter preservation was achieved in twenty-one patients (51.2%). Pathological complete response rate was 24.1%. After a median follow up of 24 months. Median PFS was 20 months and 2-years PFS was 62.3%. The median overall survival of all patients was not reached, while 2-years OS was 76.5%.

**Conclusion:**

Neoadjuvant FOLFIRINOX followed by CRT for middle and lower rectal cancer is feasible, tolerable with satisfactory sphincter preservation rate.

## Introduction

Rectal cancer is one of the major common types of gastrointestinal cancer affecting the relatively younger age group in our region, with a relatively high incidence under the age of 40 years (Veruttipong et al., 2012). Unfortunately, the treatment is usually associated with permanent colostomy when it comes in the middle or lower third of rectum (Perry et al., 2007). 

Surgery is the main line of treatment, adding postoperative radiotherapy with concurrent 5-Fluorouracil was an effective approach to decrease the rate of local recurrence (Wolmark et al., 2000). Concomitant chemoradiotherapy (CRT) is better to be given preoperative than postoperative as seen in the German CAO/ARO/AIO-94 trial, where local control was significantly better with concomitant preoperative CRT (Sauer et al., 2012). 

The addition of oxaliplatin to 5FU or to capecitabine during CRT did not show any benefit and unfortunately increased the toxicity in most of the trials (Aschele et al., 2011; Ge´rard et al., 2012; Allegra et al., 2015), with the only exception of the German trial CAO/ARO/AIO-04 (Rödel et al., 2015). Moreover, the addition of target therapy to concomitant CRT was not useful; bevacizumab in AVACROSS study was associated with postoperative complications requiring re-surgery in 24% of patients (Noggu´e et al., 2011). Additionally, cetuximab in EXPERT-C trial did not improve complete response rate (Dewdney et al., 2012). 

We were lacking data on the efficacy and safety of adding combination chemotherapy before giving concomitant CRT, especially for a very active protocol like FOLFIRINOX. FOLFIRINOX combination chemotherapy regimen consists of oxaliplatin, irinotecan, fluorouracil, and leucovorin. This regimen had a high response rate reaching 60% in metastatic colon cancer (Falcone et al., 2007). 

In order to know the value of adding FOLFIRINOX before concomitant CRT, our group in Cairo University did a phase II study to evaluate the efficacy of adding this promising regimen before concomitant CRT. Our main aim was to increase the rate of sphincter preservation, especially in patients with middle and lower rectal cancer. 

## Materials and Methods

*Patients and Methods*

This is a prospective, single arm phase II study evaluating the role of induction chemotherapy with FOLFIRINOX regimen followed by chemoradiotherapy for patients with middle and lower third rectal cancer who were presented to Kasr Al-Ainy center of clinical oncology and nuclear medicine (NEMROCK) at Cairo University. Recruitment of patients started from 1 January 2016 till 31 July 2018. The institutional research committee approved the trial protocol. The informed consent was obtained before treatment.

Sphincter preservation rate was the primary end point. Secondary end points included pathologic complete response, objective response rate, toxicity, progression free survival, and overall survival. 

*Patients*

Patients included had the following criteria; histologically proven adenocarcinoma of the rectum within the lower or middle third of rectum, the lower edge less than 10 cm from the anal verge. Patient should not have received any treatment for rectal cancer, age less than or equal to 60 years, adequate performance status of ≤ 2. While exclusion criteria included patients age above 60 years, severe active rectal bleeding, synchronous metastases with unresectable hepatic and/or lung localization, prior pelvic irradiation, symptomatic sensorimotor peripheral neuropathy, pregnant or lactating patients or patient of both sexes with childbearing potential and not using adequate contraception method, known hypersensitivity to any component of the treatment.

*Treatment*

Neoadjuvant chemotherapy consisted of four cycles of FOLFIRINOX regimen given every 2 weeks over two months. FOLFIRINOX consisted of oxaliplatin 85 mg per square meter over 2-hour intravenous infusion, followed by leucovorin 400 mg per square meter over 2-hour infusion, with the addition, after 30 minutes, of irinotecan 180 mg per square meter, given as a 90-minute intravenous infusion through a Y-connector. After that, bolus ﬂuorouracil 400 mg per square meter was given, followed by infusional ﬂuorouracil 2,400 mg per square meter over 46-hour infusion immediately after ﬂuorouracil bolus injection.

Concomitant chemoradiotherapy started from two to four weeks after completing neoadjuvant chemotherapy. Radiotherapy consisted of 50.4 Gy delivered in conventional fractionation (daily fractions of 1.8 Gy) over five and half weeks. Radiotherapy planning was divided in two phases, phase one consisted of 45 Gy in 25 fractions to whole rectum plus mesorectal fascia including tumor with 2 cm margin, internal iliac, presacral lymph nodes and without external iliac lymph nodes except in case of anterior structures involvement. Followed by phase two which is a boost to gross tumor plus surrounding mesorectal fascia plus 2 cm margin, it consisted of 5.4 Gy given in 3 fractions. Three-dimensional conformal techniques with high-energy photons (6–25 MeV) were used. Concomitant chemotherapy was delivered with bolus fluorouracil 400 mg per square meter and bolus leucovorin 20mg per square meter for 5 days during the first and fifth weeks of radiotherapy.

3D conformal radiotherapy technique using CT simulation with 5 mm thickness with prone position and belly board was used if patient underwent initial colostomy.

Surgery was performed 6 to 8 weeks after radiation treatment. Standard exploratory laparotomy with thorough examination of the intraperitoneal content was performed. The choice of surgical procedure was left to the operating surgeon. Anterior and abdominoperineal resection were all permitted. Total mesorectal excision was recommended. Pelvic organs with cancer involvement were resected en bloc if possible, to achieve an R0 resection. Adjuvant treatment started 4-6 weeks after surgery, patient started adjuvant chemotherapy for 4 months either FOLFOX4 or XELOX. 

*Study assessment*

MRI pelvis and colonoscopy were used to assess tumor response rate with use of RECIST 1.1 criteria for response evaluation by MRI. The rectal tumor response rate is either, Complete Response (CR), which defined as Disappearance of all target lesions and any pathological lymph nodes (whether target or non-target) must have reduction in short axis to < 10 mm. Partial response (PR) which equal to more than 30% decrease in sum of all target lesions in longest axis measurement. Progressive disease (PD), when progression in target disease of 20% increase in sum of longest diameter in target lesions with a 5 mm absolute increase is required or new lesion. Stable Disease (SD): Neither sufficient shrinkage to qualify for PR nor sufficient increase to qualify for PD, taking as reference the smallest sum longest diameter since the treatment started. Pathological complete response (pCR): absence of viable tumor cells in the resection specimen.

*Follow-up*

Follow-up were scheduled every 3 months. Evaluation included patient’s history, clinical examination, and blood tests. Imaging was performed every 3-6 months and when indicated. Colonoscopy was done annually and when indicated.

*Statistical analysis*

All statistics were performed using SPSS software (statistical package for social science) version 17. All data were tabulated and statistically studied by descriptive analysis as well as survival analysis in relation to different prognostic factors. The significance level was P < 0.05 .

The primary end point was sphincter preservation. Secondary end points included pathologic response, objective response rate, toxicity, progression free survival, and overall survival. Pathological complete response (pCR) means absence of viable tumor cells in the resection specimen. Common terminology criteria for adverse events CTCAE version 4.03 was used for toxicity evaluation. Progression free survival was defined as the time from entry into the study until disease progression, lost follow up or death as a result of any cause. Overall survival was calculated from the date of entry in study to date of death from any cause or lost follow up; survival analysis was done according to Kaplan-Meier method.

## Results

The median age was 41 years, ranging between 22 and 60 years. Almost half of the patients (20 patients) were below 45 years old (48.7%). Twenty-two patients were females (53.7%) and nineteen patients were males (46.3%), with a female to male ratio 1.16:1, as shown in Table 1.

**Figure 1:**
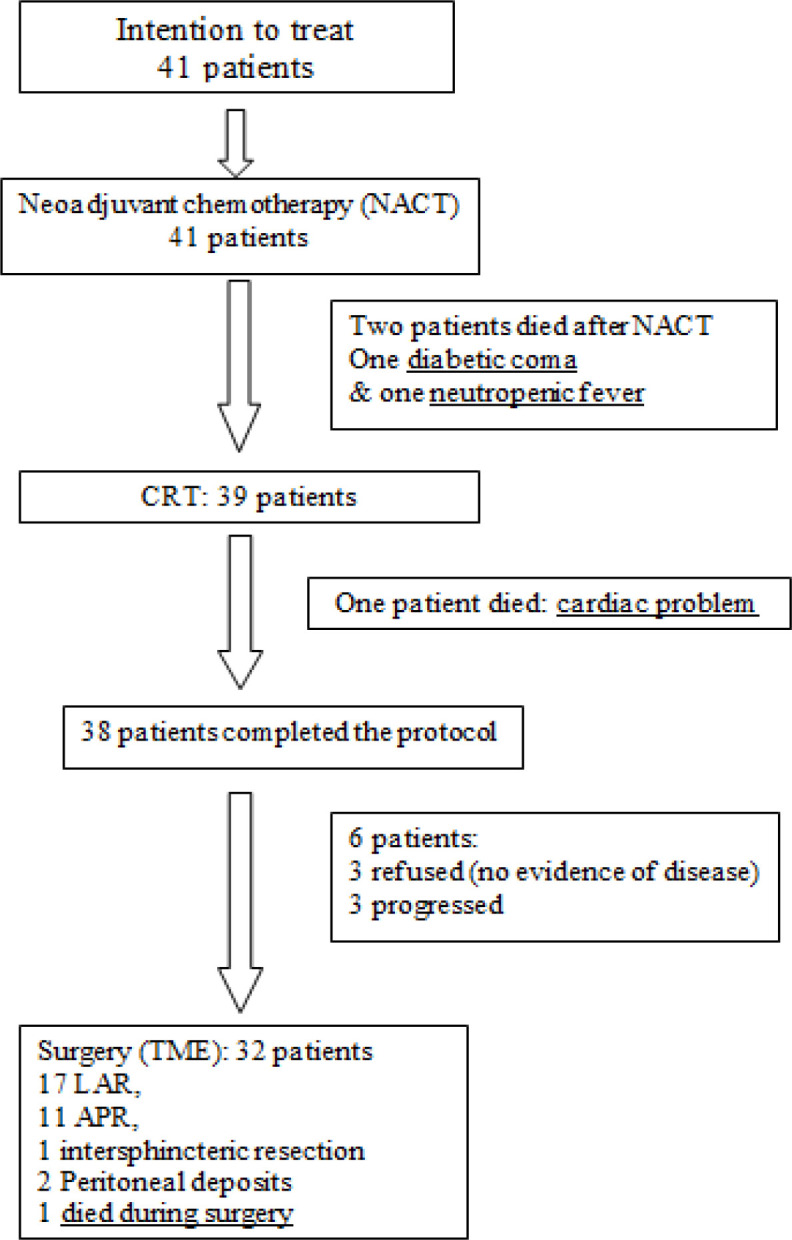
Trial algorithm. NACT, Neoadjuvant Chemotherapy; CRT, Chemoradiotherapy; TME, Total Mesorectal Excision; LAR, Low Anterior Resection; APR, Abdominoperineal Resection

All patients in our study (41 patients) completed neoadjuvant chemotherapy FOLFIRINOX regimen. Two patients died before starting neoadjuvant CRT (one died due to diabetic coma at home and the second due to neutropenic fever and septicemia). Thirty-nine patients started CRT, thirty-eight finished full course of CRT and one stopped due to cardiac troubles, treatment algorithm is shown in Figure 1. 

Out of 38 patients who ended the whole neo-adjuvant protocol, only 32 patients were referred for surgical consultation, three patients refused surgery, and three patients had documented progression (two patients progressed locally, and one patient developed distant metastasis). Out of these 32 patients, only 29 patients underwent proper surgical resection of the tumor with TME, as two patients opened, found to be metastatic by multiple small peritoneal nodules, so palliative colostomy was done, and afterwards received second line chemotherapy and one patient died during surgery thus surgical data wasn’t available. 

Out of 29 patients who underwent proper surgical resection, 17 patients underwent lower anterior resection (LAR), 11 underwent abdominoperineal resection (APR) and one underwent intersphinteric resection (transanal division of the rectum, with removal of part or the entire internal anal sphincter after TME) and one died during surgery. An R0 resection was performed in 28 patients (96.6%) and R1 resection was achieved in one patient (3.4%). Mean number of excised lymph nodes was 8.75. 

Regarding adjuvant treatment, 13 patients received 4 months XELOX, 11 patients received 8 cycles FOLFOX4, only one patient received 4 months Xeloda and 4 patients didn’t receive adjuvant treatment either they refused or delayed wound healing.

*Response rate assessment*

After neoadjuvant chemotherapy (NACT) by 2-4 weeks, thirty-nine patients were assessed by MRI pelvis, which revealed that about half of patients (19) had partial response (48.7%), complete radiological response in one patient (2.55%), stationary course in 17 patients (43.6%), while progression in two patients (5.1%).

Assessment after CRT was done using MRI pelvis and colonoscopy within 5-6 weeks after finishing CRT. Thirty-eight patients out of thirty-nine were assessed while one died due to cardiac cause during radiotherapy. In patients who were assessed after CRT complete clinical response was achieved in eleven patients (28.9%), partial response in 9 patients (23.7%), stationary course in 15 patients (39.5%), with documented progression in three patients (7.9%). 

Out of 29 patients who underwent proper surgical resection, pathological complete response (pCR) was confirmed in seven patients (24.1%). In intention-to treat analysis, pCR was achieved in ten patients (seven patients after surgery and three patients refusing surgery and were free) 24.39 %. 

Out of 11 patients who achieved cCR, 7 confirmed to have pCR, one had residual disease, while 3 refused surgery and kept under close follow up with colonoscopy and radiological assessment every 3-6 months, two of them still free, while the third patient refused follow up but still alive. In intention-to treat, sustained complete response (sCR), including pCR and sustained cCR was achieved in ten of 41 patients (24.4%). In intention to treat (ITT) analysis, Clinical response (CR or PR) was achieved in 20 out of 41 patients (48.8%). 

Sphincter preservation and tumor down staging (pathological and radiological) were assessed in thirty-two patients (twenty-nine who underwent curative surgery plus three patients who refused surgery and had cCR). Sphincter preservation was achieved in twenty-one patients (65.6 %), while downstaging was noticed in twenty-eight patients (87.5%).

In subgroup analysis, sphincter preservation was significantly higher in patients ≤ 45 years than >45 (76.5% vs. 53.3% respectively, P=0.042), in tumor located >5 cm than ≤ 5 from anal verge (92.3% vs. 47.4% respectively, P=0.0001), and the presence of mucin was associated with less sphincter preservation (40% vs. 70.4%, P=0.003) which was statistically significant, as shown in Table 2.

*Local recurrence and distant metastasis*

During follow up of 32 patients survived after surgery or who achieved cCR but refused surgery, 2-years local recurrence (either alone or with distant metastasis) had occurred in two patients (6.3%), while distant metastasis had occurred in seven (21.9%), and liver metastasis was the most common site of metastasis in four patients (12.6%) as shown in (Table 3).

*Neoadjuvant chemotherapy toxicity*

All patients in our study (41) finished neoadjuvant 2 months FOLFIRINOX. Seventeen (41.5%) of them developed anemia with G III anemia in eight patients (19.5%). Neutropenia occurred in fourteen (34.1%) with only one patient (2.4%) developed neutropenic fever. Seven patients (17.1%) developed vomiting while G III vomiting developed in only two (4.9%). Fourteen patients (34%) developed diarrhea but G IV diarrhea developed in one (2.4%). Peripheral neuropathy developed in three patients (7.3%) with grade I and II toxicity only. Toxicity details are shown in Table 4.

*Progression Free Survival and Overall Survival*

**Figure 2.**
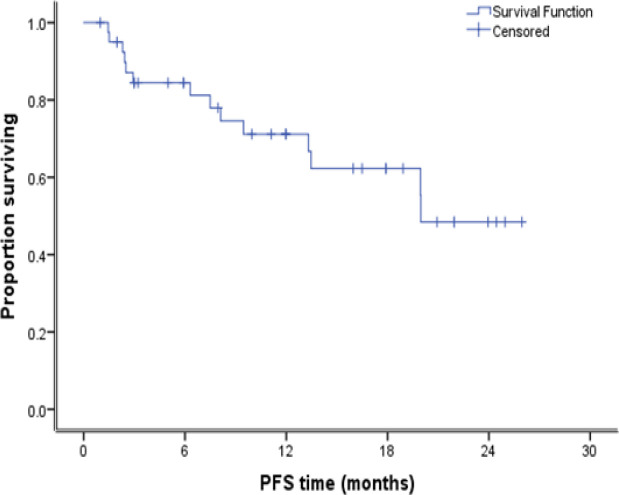
Kaplan Meier Curve for Progression Free Survival

**Figure 3:**
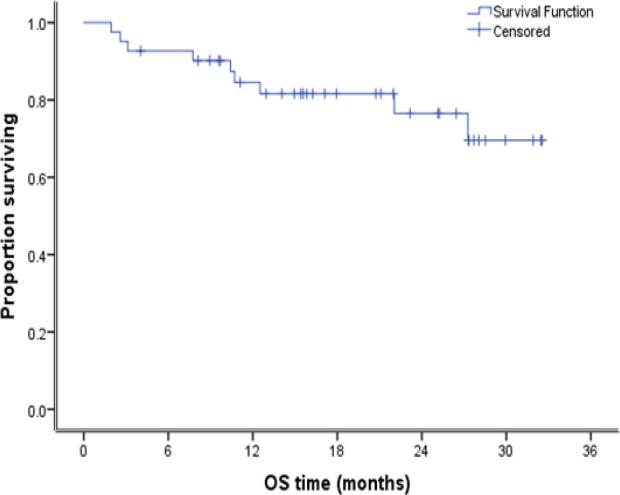
Kaplan Meier Curve for Overall Survival

After a median follow up duration of 24 months. The median PFS for all the patients was 20 months. The 2-years PFS was 62.3% as shown in Figure 2. At the time of analysis only nine patients died. Accordingly, the median overall survival of all patients was not reached. The 2-years OS was 76.5% as seen in Figure 3. 

**Table 1:** Patient Characteristics

	Number	Percentage
Age	Median 41 years, range (22-60)	
Sex		
Male	19	46.3
Female	22	53.7
Performance Status		
PS 1	31	75.6
PS 2	10	24.4
Site		
Middle	17	41.5
Lower	24	58.5
Stage		
T2N0	1	2.4
T2N+	5	12.2
T3N0	7	17.1
T3N+	23	56.1
T4N0	1	2.4
T4N+	4	9.8
Tumor length		
≤5 cm	15	36.6
>5 cm	26	63.4

**Table 2:** Subgroup Analysis for Sphincter Preservation

Factors		No		Yes		*P*-value
		N	%	N	%	
Age groups	≤ 45yrs	4	23.5	13	76.5	
	> 45yrs	7	46.7	8	53.3	0.042
Sex	Female	4	26.7	11	73.3	
	Male	7	41.2	10	58.8	0.2
Obstruction	No	10	34.5	19	65.5	0.92
	Yes	1	33.3	2	66.7	
Distant from anal verge in (cm)	≤ 5	10	52.6	9	47.4	0.0001
	>5	1	7.7	12	92.3	
Length of tumor in (cm)	≤ 5	4	28.6	10	71.4	0.37
	>5	7	38.9	11	61.1	
Pathology-Mucin	No	8	29.6	19	70.4	0.003
	Yes	3	60	2	40	
Clinically positive lymph node	Negative	3	37.5	5	62.5	0.83
	Positive	8	33.3	16	66.7	

**Table 3:** Pattern of Failure Including Local Recurrence and Distant Metastasis

Site of Failure	Number	Percentage
Local recurrence alone	1	3.1
Local recurrence with Lung & liver	1	3.1
Liver	2	6.3
Bone	2	6.3
Lung & Liver	1	3.1
Lung, Liver & Brain	1	3.1
Total	8	24.8

**Table 4:** Toxicity Profile for Neoadjuvant Chemotherapy

Adverse events	Grade 0		Grade 1		Grade 2		Grade 3		Grade 4	
	N	%	N	%	N	%	N	%	N	%
Anemia	24	58.5	1	2.4	8	19.5	8	19.5	-	-
Neutropenia	27	65.9	4	9.8	5	12.2	4	9.8	1	2.4
Vomiting	34	82.9	1	2.4	4	9.8	2	4.9	-	-
Diarrhea	27	65.9	1	2.4	5	12.2	7	17.1	1	2.4
Peripheral Neuropathy	38	92.7	1	2.4	2	4.9	-	-	-	-

## Discussion

The primary end point for most of the trials in rectal cancer is the pathological complete response rate. However, we chose the sphincter preservation rate as the primary end point as it is the usual request from most of our patients. Most of our patients are relatively in the younger age group and they requested a colostomy free life. Therefore, our strategy was directed towards treating them to become cancer free and colostomy free. Accordingly, we chose aggressive combination chemotherapy FOLFIRINOX as the upfront treatment. We considered a pathological complete response in secondary end points.

FOLFIRINOX was selected as the regimen associated with the highest response rate in metastatic colon cancer (Falcone et al., 2007). It is relatively cheaper than using target therapy, especially in a developing country, and can be tolerated in relatively younger age group patients. This is the regimen of choice in metastatic colorectal cancer, especially for patients with good performance status. We were satisfied with only 2 months duration of chemotherapy before CRT. We had 2.55% complete response rate and 48.7% partial response rate, in addition to 43.6% as stationary course to FOLFIRINOX.

For the primary end point, sphincter preservation was achieved in 51.2% of patients who underwent surgical resection or who refused surgery but kept on regular follow-up. Tumor location was the most important factor for sphincter preservation, patients with lower edge of tumor more than 5cm had a 92.3% preservation rate versus 47.4% for tumor 5cm or less from anal verge. Also, the absence of mucin and the younger age (≤ 45 years) are associated with significant higher sphincter preservation rate.

In this study, pCR was confirmed in seven patients out of 29 patients (24.1%) who underwent proper surgical resection, which is relatively better than reported in many of the large phase III trials using oxaliplatin, fluorouracil with CRT; which ranged between 13% to 19.5% only. This was seen in the German CAO/ARO/AIO-04 trial, where it was 17% in fluorouracil and oxaliplatin with CRT compared to only 13% to fluorouracil CRT group. Additionally, in the Italian randomized phase III STAR-01 trial, where it was recorded 16% in both groups (CRT with and without oxaliplatin). NSABP R-04 trial had the same reported pCR (19.5% and 17.8% for CRT with or without oxaliplatin). And even the Spanish trial GCR-3, pCR was only 14% with induction chemotherapy capecitabine and oxaliplatin followed by CRT. However, we have seen an interesting increase in percentage of pCR with consolidation neoadjuvant chemotherapy mFOLFOX6 when given after CRT, and the percentage of pCR increased by a greater number of cycles given (0 cycles 18%, 2 cycles 25%, 4 cycles 30%, 6 cycles 38%)(Garcia-Aguilar et al., 2015).

Three patients in our study underwent watch and wait policy based on their request. They were totally free by MRI imaging, endoscopy and pathological biopsy. Our main aim is to do genetic analysis for these patients with unexpectedly no evidence of disease after neoadjuvant therapy. 

We restricted the upper edge of age in our study to only 60 years old, in order to improve the toxicity profile and the tolerance of our patients to FOLFIRINOX. Even so, we had three patients that died during the neoadjuvant FOLFIRINOX and CRT. We had to monitor our patients in a better way, especially patients with cardiac problems and uncontrolled diabetic patients. On the other hand, the strategy of organ preservation is mainly directed for relatively young age group. Accordingly, in the future, we can focus on the age group below 50 years old. 

Distant metastases are still the major task in the treatment of advanced rectal cancer, despite giving FOLFIRINOX as a neoadjuvant therapy and giving adjuvant chemotherapy. Distant failure was seen in 21.9% in comparison to only 6.3% local failure. This may be explained by the relatively younger age group (median age 41 years old) and advanced stage. We can improve the outcome by increasing the number of cycles of neoadjuvant chemotherapy and performing genetic analysis for those patients with aggressive metastatic behavior. 

The opposite sequencing approach in total neoadjuvant therapy by starting CRT followed by chemotherapy and then surgery is very promising. In the recently published randomized phase II German CAO/ARO/AIO-12 trial where pCR was significantly better for the new sequencing (CRT followed by chemotherapy) 25% versus 17% for chemotherapy followed by CRT (P <0.001). As the compliance to CRT is better, the compliance to chemotherapy was worse (Fokas et al., 2019). We are waiting for long-term follow-up results of this trial to know how the real value of improved pCR can affect the other parameters like local recurrence, disease free survival and overall survival. In addition, phase III CAO/ARO/AIO-18 can give us more accurate results.

We had only a median of 24 months follow up period, which is relatively short to know the overall survival. Full data on mature overall survival will be published in the future. However, 2-years OS was 76.5%. This is relatively less than CAO/ARO/AIO-94 (5-year OS 76%-74%), CAO/ARO/AIO-04 (3-years OS 88%-88.7%), ACCORD 12/0405 PRODIGE 2 trial (3-years OS 88.3%-87.6%), and NSABP R-04 trial (5-year OS 80.8%-79.9%). Again, this is can be explained by the aggressive behaviour of rectal cancer in our region with relatively high incidence of distant metastases.

We recognize that this trial had some limitations. We did not search for biomarkers like RAS, BRAF, MSI or UGT1A1, in addition to having a relatively small number of patients included. However, this pilot study proved the efficacy of FOLFIRINOX in advanced rectal cancer.

In conclusion, this trial confirmed that neoadjuvant FOLFIRINOX followed by CRT for middle and lower rectal cancer is feasible and tolerable with satisfactory sphincter preservation rate. It can be an option to start a phase III trial of total neoadjuvant treatment, especially when sphincter preservation is requested.
